# Vestibular function tests are helpful in differentiating between Menière’s disease and vestibular migraine

**DOI:** 10.3389/fneur.2025.1569247

**Published:** 2025-08-07

**Authors:** Eleonora G. M. Vosbeek, Meinie Seelen, Aliede M. Vingerhoed, Tjard R. Schermer, Suzanne C. Cannegieter, Gisela M. Terwindt, Tjasse D. Bruintjes

**Affiliations:** ^1^Apeldoorn Dizziness Centre, Gelre Hospitals, Apeldoorn, Netherlands; ^2^Department of Otorhinolaryngology – Head and Neck Surgery, Leiden University Medical Center (LUMC), Leiden, Netherlands; ^3^Department of Primary and Community Care, Radboudumc Research Institute for Medical Innovation, Radboud University Medical Centre, Nijmegen, Netherlands; ^4^Department of Clinical Epidemiology, Leiden University Medical Center (LUMC), Leiden, Netherlands; ^5^Department of Internal Medicine, Division of Thrombosis and Haemostasis, Leiden University Medical Center (LUMC), Leiden, Netherlands; ^6^Department of Neurology, Leiden University Medical Center (LUMC), Leiden, Netherlands

**Keywords:** caloric test, video head impulse test, audiometry, diagnosis, vertigo, headache, dissociation, catch-up saccades

## Abstract

**Aim:**

Menière’s disease (MD) presents with episodic vertigo and auditory symptoms. Vestibular migraine (VM) typically contains migraine features associated with the vertigo attacks. Distinguishing MD from VM can be challenging due to overlap in symptomatology. To assist in the differentiation between the two, this study aimed to compare auditory and vestibular symptoms and functions in MD and VM, and to assess the diagnostic value of the video head impulse test (vHIT) and caloric test.

**Methods:**

A cohort study was performed at a tertiary dizziness clinic in the Netherlands in MD and VM patients seen in our clinic from January 2018 until September 2024. Patients were diagnosed based on the Bárány Society criteria. We collected demographic characteristics, symptoms at presentation, results of pure-tone audiometry (PTA), caloric testing, and vHIT. Positive predictive value (PPV) and negative predictive value (NPV) of the vHIT and caloric test were calculated and the optimal unilateral weakness cut-off value was determined.

**Results:**

The mean age of the MD patients (*n* = 194) was 60.2 ± 13.4 years, with 46.4% females. The VM patients (*n* = 101) had a mean age of 50.2 ± 14.6 years, with 86.1% females. Not only MD patients, but also 65.7% of VM patients experienced at least one aural symptom during vertigo attacks. An abnormal caloric test, abnormal horizontal vHIT, and catch-up saccades during the vHIT were observed more frequently in MD than in VM patients. The vHIT had a PPV of 81% and an NPV of 36% to distinguish MD from VM. Additionally, in patients with a normal vHIT, the caloric test had a PPV of 82% with an NPV of 55%. Increasing the unilateral caloric weakness threshold to 34%, increased the PPV to 90%, with an NPV of 52%.

**Conclusion:**

While the diagnosis of MD and VM is based on history and audiometry findings, vHIT and caloric testing may aid in differentiating between the two diseases in ambiguous cases. If either the vHIT or caloric test is abnormal, a diagnosis of MD is more likely. The optimal PPV and NPV to differentiate between MD and VM was found with a unilateral caloric weakness threshold of 34%.

## 1 Introduction

The differential diagnosis in patients with vertigo attacks can be challenging but is essential because of different prognosis and therapeutic options. Menière’s disease (MD) is a multifactorial vestibular disorder that is characterized by episodes of vertigo, which may manifest in various ways (1). It is typically associated with aural symptoms such as hearing loss, tinnitus and aural fullness (2). Vestibular migraine (VM) is a subtype of migraine, also presenting with vertigo attacks, but needs to be accompanied by migrainous headache, photo- and phonophobia, or visual aura in at least half of episodes according to the International Classification of Headache Disorders third edition (ICHD-3) (3, 4). VM may mimic MD and vice versa, especially in early stages of these diseases. In MD, hearing loss may not occur until later in the course of the disease (2). Furthermore, it has been described that patients with VM may also develop hearing loss, although this does not progress to profound levels (5, 6).

The international diagnostic criteria for MD (2) and VM (4) are mainly based on the patient’s history. However, it has been suggested that audiological and vestibular testing may aid in differentiating between MD and VM, as well as ruling out other vestibular disorders (7), especially when the patient does not fully meet the diagnostic criteria. Vestibular function tests such as the caloric test and the video head impulse test (vHIT) can reveal dysfunction of the peripheral vestibular system (7). The use of vestibular testing in differentiating between MD and VM has been studied before (6, 8–31). In these studies, caloric hypofunction was found in up to 28% of VM patients, compared to percentages up to 71% in MD patients (8, 19, 27). Furthermore, a dissociation between the results of vHIT and caloric testing, meaning a normal vHIT combined with an abnormal caloric test result, has been described as a typical feature for MD by some (8, 10, 24, 32, 33). The presence of this dissociation was seen in up to 58.9% of MD patients, but was also observed in other, mainly peripheral, lesions (8, 34). An explanation of this interesting phenomenon observed in MD may be an increased diameter of the hydropic semicircular duct, which minimally affects the rotational response, but is thought to reduce the thermally induced pressure elicited by caloric stimulation (33). This suggests a frequency-dependent impairment of canal function in MD (35). Unfortunately, interpretation of previous studies is hampered due to small sample sizes, outdated diagnostic criteria (9–13, 15, 25, 31), unclear criteria (26), or inclusion of probable MD and VM cases which results in an underestimation of the true difference between groups.

This study aimed to compare auditory and vestibular symptoms and functions in MD and VM, while also evaluating the diagnostic value of the vHIT and caloric test to aid in distinguishing between the two conditions.

## 2 Methods

### 2.1 Study design and setting

An observational cohort study was performed at the Apeldoorn Dizziness Centre, a tertiary dizziness clinic in the Netherlands. Data from all consecutive adult patients who attended an intake visit at the outpatient clinic and were diagnosed with MD or VM between January 2018 and September 2024 were gathered retrospectively from the electronic patient files.

### 2.2 Study population

Patients aged 18 years or older at the moment of vestibular testing at our tertiary dizziness clinic, were included if they fulfilled the criteria of the International Classification of Vestibular Disorders (ICVD) consensus documents for MD (2) and/or VM (4), which are supported by the Bárány Society. The diagnostic classification according to these criteria was performed by two researchers (EV and AV). The following diagnosis groups were formed: definite unilateral MD (MD), probable MD (pMD), definite VM (VM), and probable VM (pVM). Separate diagnostic categories were formed for patients fulfilling both definite MD and VM criteria (MDVM), patients fulfilling both pMD and pVM criteria (pMDpVM), and those fulfilling bilateral MD criteria, to exclude them from the analyses. All past audiometric results, up to the initial consultation at the study center, were reviewed. At least one pure tone audiogram with asymmetrical low-frequency sensorineural hearing loss was necessary for the diagnosis of definite MD. For a diagnosis of definite VM, a current or past diagnosis of migraine (with or without aura) according to the ICHD-3 criteria was required, in line with the ICHD-3 Appendix criteria for VM (3). Only those patients with clearly documented migrainous headaches were classified as VM, or MDVM, while those with non-migrainous headaches or those lacking sufficient headache characterization were not assigned a VM diagnosis. Exclusion criteria were prior intratympanic (IT) gentamicin injection, prior endolymphatic duct surgery, vestibular dysfunction due to etiologies other than MD or VM (e.g., cerebellar pathology or vestibular neuritis), and vestibular schwannoma. Patients were also excluded in case of absent vestibular test reports and in case they had objected against the use of their medical record data for research purposes.

### 2.3 Data collection

Demographic characteristics, history of migraine, presenting symptoms, results of pure-tone audiometry (PTA), caloric test results and vHIT results were collected. Sex was determined based on the hospital registration records, where individuals were identified as male or female. Data were collected by a PhD student (EV), a medical student (AV) and a biomedical student. Dubious cases were discussed with an otorhinolaryngologist and a neurologist and a diagnosis was established based on consensus.

### 2.4 Audiometric and vestibular tests

All audiometric and vestibular tests for each patient were performed on the same day and in an interictal period.

Hearing test results used in the analyses were derived from the pure tone, air conducted thresholds bilaterally at 500, 1,000, 2,000, 4,000 and 8,000 Hz, performed at the initial consultation at our dizziness clinic. Low Fletcher Index was defined as average hearing thresholds at 500, 1,000 and 2,000 Hz (36). High Fletcher Index was defined as average hearing thresholds at 1000, 2000 and 4,000 Hz (37). Overall hearing loss was defined as a mean pure tone threshold ≥35 dB HL at 500, 1,000, 2,000, and 4,000 Hz in at least one ear. This definition was used to describe the general presence of hearing impairment and does not necessarily indicate unilateral or MD-specific hearing loss. To assess for asymmetrical low-frequency hearing loss suggestive of Meniere’s disease, we also calculated interaural differences. Asymmetry was defined as a ≥ 30 dB difference between ears at 500 and 1,000 Hz, in accordance with the Bárány criteria for MD-related hearing loss (2).

Conventional open loop bithermal (33 ⁰C and 44 ⁰C) caloric testing with water irrigation was performed, using the ATMOS Variotherm Plus device and a video-based system (HORTMANN Vestlab 7.0, Otometrics, Germany and since 2023 using the ICS Impulse, OTOsuite Vestibular software: Natus-Otometrics, Taastrup, Denmark) to record the ocular response. During testing, subjects were in a supine position with a head elevation of 30⁰. Each irrigation took place during an interval of 35 s. Unilateral weakness (UW) was defined according to the Jongkees equation (38). The caloric test response was considered abnormal when UW was ≥22% (39).

The vHIT was performed using the commercially available mono-ocular video oculography system of ICS Impulse (OTOsuite Vestibular software: Natus-Otometrics, Taastrup, Denmark). Equal to the description by van Esch et al. (40), subjects were instructed to maintain fixation at a dot from 1 to 3 m distance. An experienced vestibular technician delivered head impulses (10⁰-20⁰ angle, duration 150–200 ms, peak velocity of >150 ⁰/s) in the horizontal plane with unpredictable timing and direction. The vestibulo-ocular reflex (VOR) gain was defined as the ratio of the mean eye velocity (⁰/s) to the mean head velocity (⁰/s). The presence of catch-up saccades, defined as consistent overt or covert saccades following head impulses, was evaluated by the vestibular technician through interpretation of the eye and head velocity traces. In case of an inconclusive report, an independent clinical audiologist was consulted. An abnormal horizontal vHIT was defined as a gain <0.8, with the presence of ipsilateral catch-up saccades indicating an abnormal VOR (13, 15, 40, 41).

### 2.5 Statistical analysis

Normal distribution of continuous variables was first checked visually, followed by assessing skewness and kurtosis, and statistical testing of normality using the Shapiro–Wilk test. For demographic variables and symptoms, means and standard deviations, or medians and interquartile ranges in the case of non-normally distributed data, were calculated. Dichotomous demographic variables and vestibular function test results were reported as number and percentage. Differences between the diagnosis groups were first assessed by means of cross-tabulation and analyzed using the Chi-square test. Differences in age, duration of symptoms, and continuous variables of audiograms and vestibular test results between MD, pMD, VM and pVM were analyzed using the ANOVA or Kruskal-Wallis test, the latter in case of non-normally distributed data. A *p*-value of <0.05 was considered statistically significant. To assess the diagnostic value of the vHIT and the caloric test to differentiate MD from VM, the sensitivity, specificity, positive predictive value (PPV) and negative predictive value (NPV) were calculated. By means of the receiver operating characteristic (ROC) curve and Youden’s index, the optimal cut-off value for the caloric test was determined.

Due to the explorative nature of this study, no sample size was calculated and no correction for multiple testing was applied. The statistical analyses were independently verified by a second researcher (TS).

### 2.6 Ethical approval

This study was conducted in accordance with the World Medical Association’s Declaration of Helsinki. The Institutional Review Board of Gelre Hospitals Apeldoorn and Zutphen judged the study as not being subject to the Dutch Medical Research Involving Human Subjects Act (‘WMO’), and permission was granted to refrain from obtaining patients’ informed consent (file number 2024_02).

## 3 Results

### 3.1 Demographics and history

**Figure 1:**
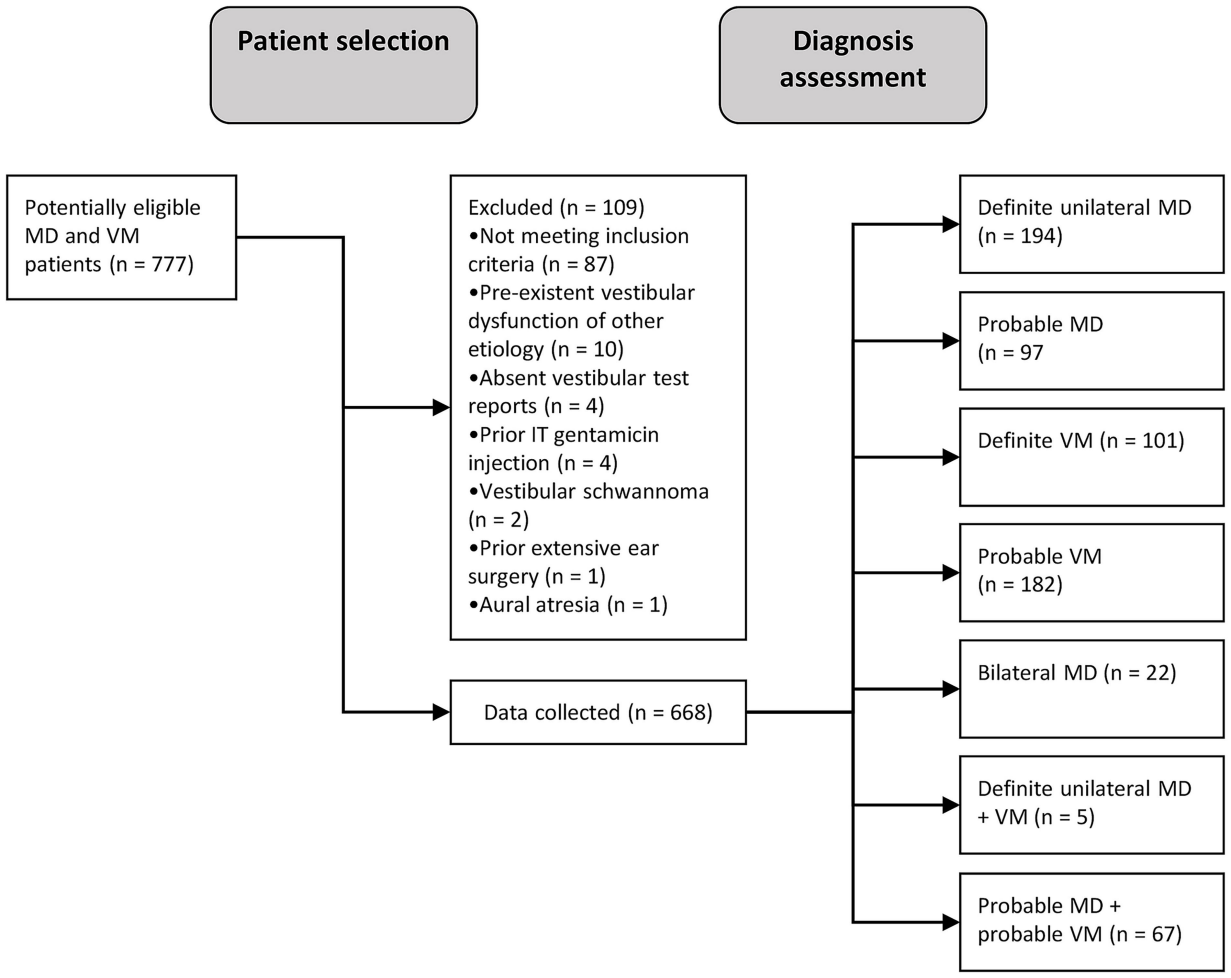
Flow diagram showing patient selection and -inclusion process. MD, Menière’s disease; VM, vestibular migraine; n, number of patients; IT, intratympanic.

We included a total of 668 patients diagnosed with Menière’s disease and/or vestibular migraine patients, of whom 194 fulfilled unilateral MD criteria, 97 pMD criteria, 22 bilateral MD criteria, 101 VM criteria and 182 pVM criteria (Figure 1). Patients fulfilling the criteria of two diagnoses were placed in the pMDpVM group (*n* = 67), and in the MDVM group (*n* = 5), and these overlap groups were excluded from the analyses. The VM group consisted of 86.1% females, as opposed to 46.4% in the MD group (Table 1). The median duration of symptoms at the time of inclusion was 4.0 years in the MD, VM and pVM groups, and 2.0 years in the pMD group. In the bilateral MD patients and MDVM patients, the duration was longer, namely a median of 19.0 years and 20.0 years, respectively (Supplementary Table 1).

**Table 1:** Demographics and history of migraine in MD, pMD, VM, and pVM patients.

Diagnosis	MD *n* = 194	pMD *n* = 97	VM *n* = 101	pVM *n* = 182	*p*-value^a^
Sex, *n* female (%)	90 (46.4%)	57 (58.8%)	87 (86.1%)	138 (75.8%)	<0.001
Age at presentation, years (mean±SD)	60.2 ± 13.4	58.8 ± 14.7	50.2 ± 14.6	54.1 ± 15.3	<0.001
Duration of symptoms, years (median (IQR))	4.0 (9.0) *n* = 187	2.0 (4.0) *n* = 92	4.0 (8.5) *n* = 89	4.0 (10.0) *n* = 164	0.002^b^
History of migraine, *n* (%)	32 (17.1%) *n* = 187	2 (2.1%) *n* = 94	101 (100.0%) *n* = 101	84 (46.7%) *n* = 180	<0.001

a *p*-value given for the comparison of MD, pMD, VM, and pVM.

b *Post-hoc* pairwise comparisons with the Mann–Whitney U test revealed significant differences regarding the pMD patients; between MD and pMD (*p* < 0.001), pMD and VM (*p* = 0.010), and pMD and pVM (*p* < 0.001).

MD, definite unilateral Menière’s disease; pMD, probable Menière’s disease; VM, definite vestibular migraine; pVM, probable vestibular migraine; n, number of patients; SD, standard deviation; IQR, interquartile range.

### 3.2 Symptoms

**Figure 2:**
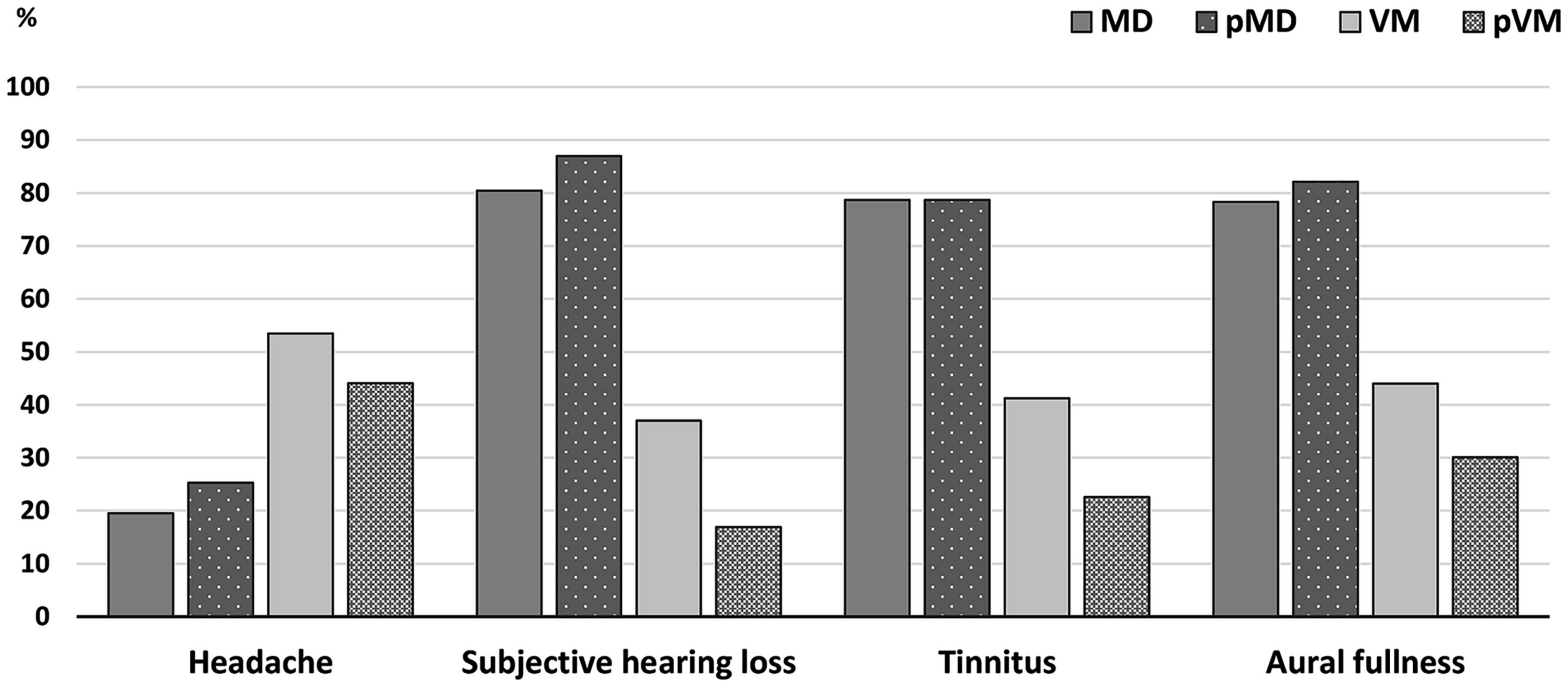
Bar graphs comparing ictal symptoms in MD, pMD, VM, and pVM patients. Presence of ictal headache 19.5% (MD); 25.3% (pMD); 53.5% (VM); 44.1% (pVM). Presence of ictal subjective hearing loss: 80.4% (MD); 87.0% (pMD); 37.0% (VM); 16.9% (pVM). Presence of ictal tinnitus: 78.7% (MD); 78.7% (pMD); 41.2% (VM); 22.6% (pVM). Presence of ictal aural fullness: 78.3% (MD); 82.1% (pMD); 44.0% (VM); 30.1 (pVM). *p*-value for the comparison of MD, pMD, VM, and pVM reached <0.001 for each symptom. MD, definite unilateral Menière’s disease; pMD, probable Menière’s disease; VM, definite vestibular migraine; pVM, probable vestibular migraine.

Ictal headache occurred in 53.5% of the VM patients, but also in 19.5% of MD patients and 25.3% of pMD patients, where it specifically involved non-migrainous headache (Figure 2). Subjective hearing loss, tinnitus and aural fullness were more common in MD patients compared to VM patients (all group comparisons *p* < 0.001, Figure 2). As many as 65.7% of VM patients experienced at least one ictal aural symptom, whereas this was 100.0% in MD and pMD patients. We did not distinguish between unilateral and bilateral symptoms in these results. In the VM group, aural fullness was the most common aural symptom (44.0%, Figure 2).

### 3.3 Audiometry test results

**Figure 3:**
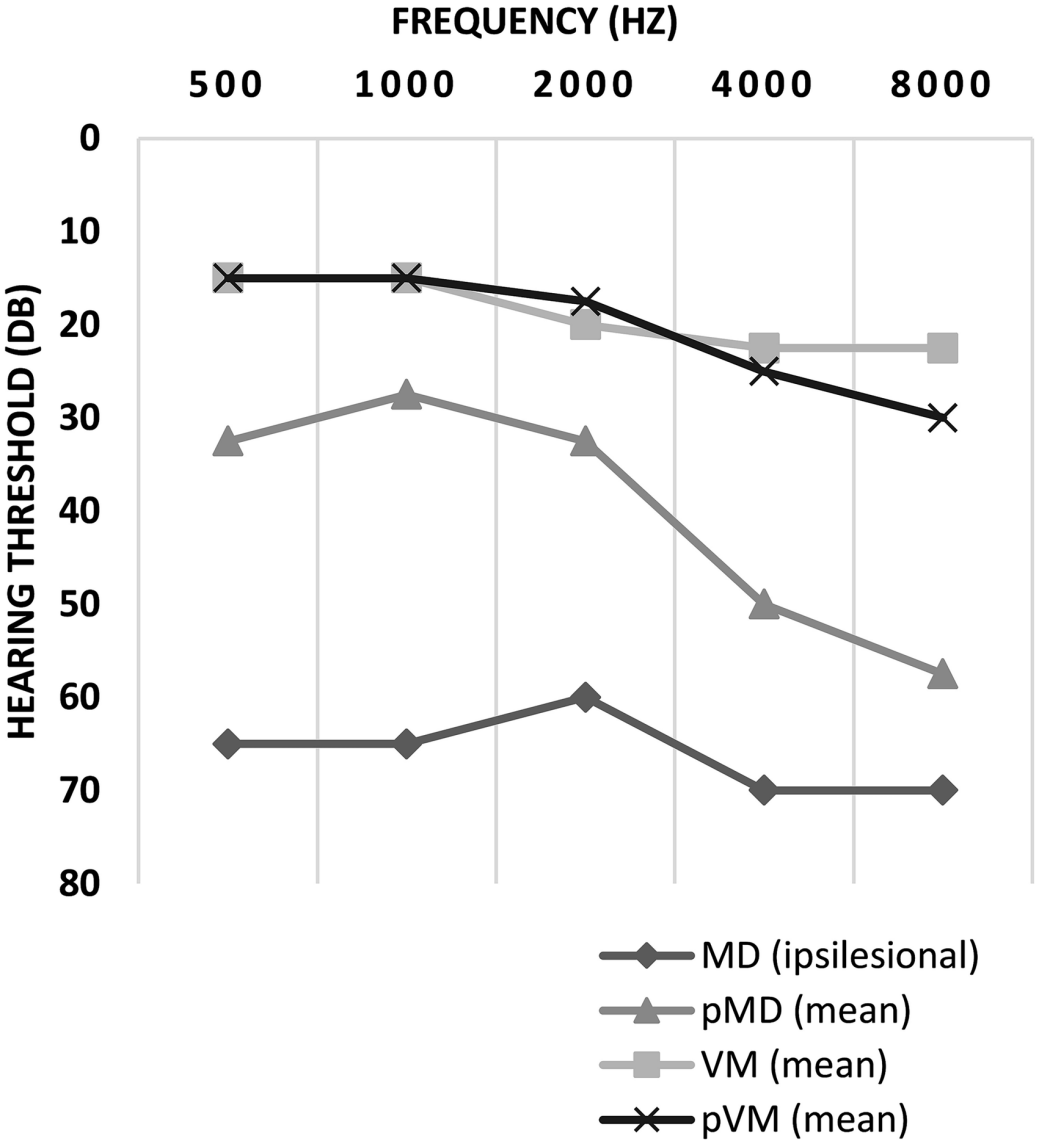
Auditory thresholds in ipsilesional MD, pMD, VM and pVM patients. For MD patients, thresholds of the ipsilesional side are displayed. For pMD, VM and pVM patients the mean of both sides is displayed. Thresholds are displayed as medians. MD, definite unilateral Menière’s disease; pMD, probable Menière’s disease; VM, definite vestibular migraine; pVM, probable vestibular migraine.

Auditory thresholds in VM and pVM patients were similar, but MD patients showed much higher thresholds for all frequencies (Figure 3). Asymmetrical low-frequency hearing loss was rarely present in VM and pVM patients (2.0 and 3.4%, respectively, Table 2), in whom no aural symptoms were present. When considering hearing thresholds at all frequencies, nearly one quarter of all VM patients showed overall hearing loss (24.2%).

**Table 2:** Pure-tone audiometry results in MD, pMD, VM, and pVM patients.

Diagnosis	MD ipsilesional side *n* = 191	MD contralesional side *n* = 191	pMD *n* = 98	VM *n* = 99	pVM *n* = 179
Low FI^a^, dB (median (IQR))	62.0 (19.0)	20.0 (16.0)	31.0 (28.6)	17.0 (14.0)	16.0 (15.5)
	MD		pMD	VM	pVM
Asymmetrical low-frequency hearing loss^b^, *n* (%)	190 (100.0%) *n* = 190^c^		0 (0.0%) *n* = 93	2 (2.0%) *n* = 99	6 (3.4%) *n* = 179
Overall hearing loss^d^, *n* (%)	181 (94.8%)		62 (66.7%)	24 (24.2%)	45 (25.1%)

a For probable MD, VM, and probable VM patients, the mean of both sides is presented.

b Pure tone threshold difference ≥30 dB between both ears at 500 and 1,000 Hz.

c One MD patient was excluded from this result due to otosclerosis on the contralesional side.

d Mean pure tone threshold at 500, 1000, 2000 and 4,000 Hz ≥35 dB, at least on one side.

MD, definite unilateral Menière’s disease; pMD, probable Menière’s disease; VM, definite vestibular migraine; pVM, probable vestibular migraine; FI, Fletcher Index; dB, decibels; IQR, interquartile range; *n*, number of patients.

### 3.4 Vestibular function test results

**Figure 4:**
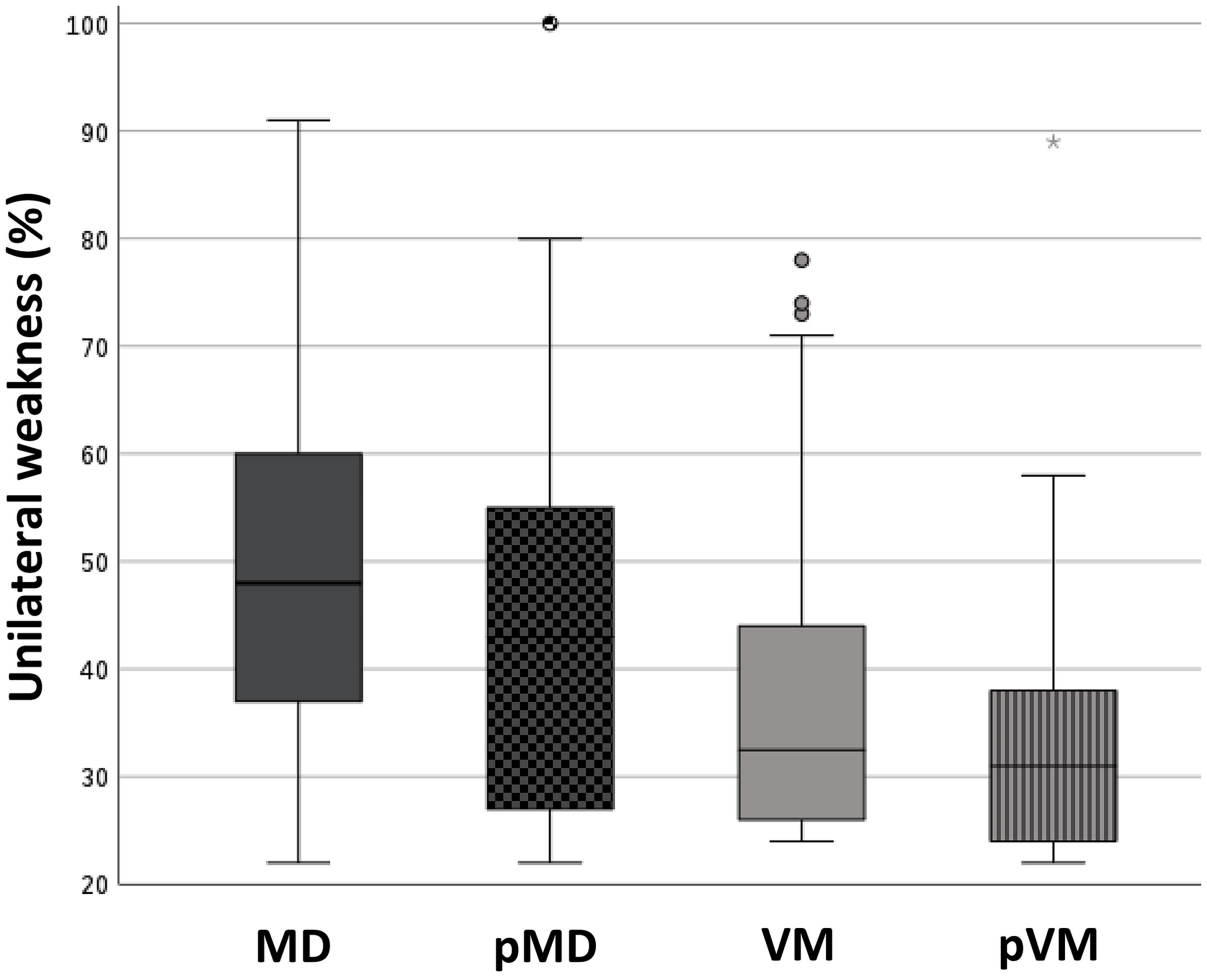
Box plots showing the degree of unilateral weakness in patients with an abnormal caloric test result. Median (IQR): 48.0% (23.0%) (MD); 43.0% (28.0%) (pMD); 33.0% (19.0%) (VM); 31.0% (14.0%) (pVM). Kruskal-Wallis test revealed a *p* < 0.001 for the comparison of MD, pMD, VM, and pVM. Post-hoc pairwise comparisons with Mann–Whitney U test revealed significant differences between MD and VM (*p* = 0.002), MD and pMD (*p* = 0.048), MD and pVM (*p* < 0.001), and pMD and pVM (*p* = 0.014). MD, definite unilateral Menière’s disease; pMD, probable Menière’s disease; VM, definite vestibular migraine; pVM, probable vestibular migraine.

Abnormal caloric test results were seen in two thirds of MD patients (66.3%), but were also seen in 28.0% of those affected by VM (*p* < 0.001, Table 3). Median UW in patients with an abnormal caloric test result was 48.0% in MD, and 33.0% in VM (*p* = 0.002, Figure 4). Only 15.3% of MD patients demonstrated abnormal horizontal vHIT results (Table 3). Catch-up saccades during the vHIT were more often seen on the ipsilesional side of MD patients, when compared to VM patients (28.9% versus 12.0%, *p* = 0.001, Table 3). A caloric-vHIT dissociation, meaning a normal vHIT result combined with an abnormal caloric test result, was seen in 56.5% of MD patients, and in 24.2% of VM patients.

**Table 3:** Vestibular function test results showing abnormal caloric test, abnormal horizontal vHIT, and catch-up saccades during the vHIT in MD, pMD, VM, and pVM patients.

Diagnosis	MD		pMD	VM	pVM	*p-value*
Abnormal horizontal vHIT^a^, *n* (%)	29 (15.3%) *n* = 190		10 (10.6%) *n* = 94	7 (7.1%) *n* = 99	8 (4.6%) *n* = 173	0.005^b^
Catch-up saccades^c^, *n* (%)	Ipsilesional 54 (28.9%) *n* = 187	Contralesional 17 (9.1%) *n* = 187	16 (17.0%) *n* = 94	12 (12.0%) *n* = 100	19 (10.7%) *n* = 177	<0.001^d^
Abnormal caloric test^e^, *n* (%)	118 (66.3%) *n* = 178		50 (54.3%) *n* = 92	26 (28.0%) *n* = 93	27 (16.6%) *n* = 163	<0.001^f^

a Gain <0.8 and presence of ipsilateral catch-up saccades.

b *Post-hoc* pairwise comparisons with the Chi-square test revealed significant differences between MD and VM (*p* = 0.045), and MD and pVM (*p* < 0.001).

c Catch-up saccades during the vHIT for pMD, VM, and pVM patients were labeled as present in case of presence on at least one, but any, side.

d *p*-value given for the comparison of ipsilesional catch-up saccades in MD, compared with any catch-up saccades in VM, pMD and pVM. *Post-hoc* pairwise comparisons with the Chi-square test revealed significant differences between MD and VM (*p* = 0.001), MD and pMD (*p* = 0.030) and MD and pVM (*p* < 0.001).

e UW ≥ 22%.

f *Post-hoc* pairwise comparisons with the Chi-square test revealed significant differences between MD and VM (*p* < 0.001), MD and pVM (*p* < 0.001), pMD and VM (*p* < 0.001), pMD and pVM (*p* < 0.001), and VM and pVM (*p* = 0.030).

MD, definite unilateral Menière’s disease; pMD, probable Menière’s disease; VM, definite vestibular migraine; pVM, probable vestibular migraine; *n*, number of patients; vHIT, video head impulse test.

### 3.5 Diagnostic value of vestibular tests to differentiate MD from VM

The PPV of the vHIT was 81% for distinguishing MD from VM (Table 4), indicating that an abnormal vHIT result corresponds to an 81% likelihood of an MD diagnosis as compared to VM. Conversely, the NPV was 36%, reflecting the limited ability of a normal vHIT to exclude MD. Performing the caloric test in all patients with a normal vHIT resulted in a PPV of 82%, with an NPV of 55%. The optimal cut-off value for UW in separating MD from VM, in case of a normal vHIT, was derived from the ROC curve and Youden’s index, and was determined to be 34%, with a sensitivity of 53% and a specificity of 90%, corresponding with a PPV of 90% and an NPV of 52% (Table 4).

**Table 4:** Diagnostic values of vHIT and caloric testing to differentiate MD from VM.

Diagnostic values	PPV, %	NPV, %	Sensitivity, %	Specificity, %
vHIT^a^	81%	36%	15%	93%
Caloric test (UW ≥ 22%)^b^	82%	55%	65%	75%
Caloric test (UW ≥ 34%)^b^	90%	52%	53%	90%

MD was analyzed as the reference group.

a Abnormal horizontal vHIT was defined as a gain <0.8, with the presence of ipsilateral catch-up saccades.

b Only patients with a normal vHIT were included in these calculations.

vHIT, video head impulse test; MD, definite unilateral Menière’s disease; VM, definite vestibular migraine; PPV, positive predictive value; NPV, negative predictive value.

## 4 Discussion

vHIT and caloric testing can help differentiate MD from VM in unclear cases, though diagnosis primarily relies on history and audiometry. An abnormal vHIT or caloric test suggests a higher likelihood of MD. The best predictive value for distinguishing MD from VM was achieved with a unilateral caloric weakness threshold of 34%.

### 4.1 Overlap and differentiation between MD and VM

Our data show a considerable overlap in symptomatology between MD and VM patients. We found that more than half of our clinically diagnosed VM patients also experienced subjective hearing loss, tinnitus, or aural fullness, symptoms which are usually assigned to MD. This may also indicate misdiagnosed cases, as patients could be in the early stage of MD, where the audiometry test can still be normal. As expected, we saw a female preponderance in VM, in accordance with what is known from previous studies and what is known about migraine in general, i.e., up to a 5 to 1 female–male ratio (42).

Based on our results, the vHIT and caloric test may be helpful in patients in whom there is diagnostic uncertainty between MD and VM. An abnormal vHIT result was observed both in MD and VM, but corresponded with a PPV of 81% to predict MD. Considering an NPV of 36%, however, in those with a normal vHIT result, the caloric test becomes an important complementary tool. An upper normal limit of UW at 34% yielded a PPV of 90%, making the diagnosis MD more likely in cases with pronounced UW. Furthermore, looking at the pMD and pVM groups in our study, the percentages of abnormal vHIT and caloric test results are quite similar to the definite MD and VM groups, respectively. Based on these findings, we propose that these vestibular test results may guide diagnostic decisions in patients in whom there is doubt between the diagnoses MD and VM. An abnormal vHIT or caloric test result is not specific for a diagnosis of MD, but such findings make a diagnosis of MD more likely than VM. Notably, the optimal sensitivity and specificity for separating MD from VM was achieved with a UW threshold of 34%.

### 4.2 Comparison with earlier studies

The 66% prevalence rate of vestibular unilateral weakness in our MD patients is similar to the rates reported in other studies in which the caloric test was compared between MD and VM patients (9–12, 19, 26, 41). We found a much lower percentage of unilateral weakness in VM patients compared to MD patients, but still 28% of VM patients had a vestibular hypofunction. This is also similar to most of the results published earlier (10–12, 19, 41), but some studies have reported substantially lower rates (27, 43), again emphasizing the difficulties in correctly diagnosing these paroxysmal disorders. We found that in case of a normal vHIT result, the caloric test has a high PPV for a diagnosis of MD, which is in concordance with the diagnostic value of the dissociation between the vHIT and caloric test in MD, as described by others (8). Interestingly, in patients with an abnormal caloric test, the hypofunction was more severe in MD than VM, which has previously been described by Yilmaz et al. (31). Therefore, our result that the optimal UW threshold to differentiate between MD and VM is 34%, makes sense. Comparable to earlier studies, we observed more catch-up saccades during the vHIT in our MD population than in the VM group (18, 27, 31).

We propose that the primary focus should not be on the dissociation between the vHIT and caloric test as a diagnostic marker for MD, as has been outlined before (8, 10, 24, 32, 33), but rather on the fact that in patients with recurrent vertigo and evident vestibular loss, the diagnosis of MD is highly likely. In such cases, performing both the vHIT and caloric test is not always necessary. Since the vHIT is often sufficient to detect abnormalities in these situations, the caloric test can consequently be considered redundant (40).

Seventeen percent of our MD patients had a history of migraine and nineteen and a half percent experienced ictal headache. Our numbers are lower than the rates of migraine of 50–56% in MD patients reported by other authors (20, 44, 45). In fact, our numbers are comparable to the estimate of the global migraine prevalence of 14–15% (46). A possible explanation might be that the diagnosis of VM was previously less recognized. Consequently, the MD groups in older studies may have included patients who, with the current criteria, would better fit into the VM category, potentially skewing the prevalence of characteristics of both conditions. We did not analyze data on visual aura and photo- or phonophobia, but it would be interesting to include these VM features in future research comparing MD and VM patients.

### 4.3 Strengths and limitations

Strengths of our study are the relatively large patient sample, and the use of the most recent, internationally accepted diagnostic criteria for MD and VM, the diagnostic gold standard. Furthermore, we made a distinction between MD, VM, pMD, pVM, bilateral MD, and two overlap groups, namely MDVM and pMDpVM. By categorizing the MD group in MD and pMD and the VM group in VM and pVM for all outcomes, we enhance the interpretability of our results. Applying these findings to patients with an uncertain diagnosis is more reliable than relying on findings from groups that include a mix of patients with both definite and probable diagnoses.

We observed no significant difference in disease duration between the MD and VM groups. We therefore expect that our results remain unaffected by the assumption that the degree of vestibular dysfunction is correlated with the stage of MD, as has been demonstrated in previous studies (23, 47).

Our results may have been influenced by selection bias, since we studied a population which, partially, consists of tertiary referrals, and the execution of the study in a single center may impair generalizability to a wider population. It is also important to note that the predictive values of the vHIT and caloric test mentioned in this study are specific to a population consisting solely of MD and VM patients. Therefore, these findings cannot be directly extrapolated to a broader population of patients with dizziness in general. Lastly, although the diagnostic classification of patients was conferred by strictly adhering to the criteria, the investigators were not blinded for the available clinical information, including vestibular test results, which may have influenced the diagnostic process.

## 5 Conclusion

In conclusion, knowledge about the differences and similarities with regards to demographic features, patient history, symptoms, and vestibular function between MD and VM will assist clinicians in diagnosing dubious cases. Although our study focused on patients with a diagnosis of VM or MD, our findings suggest that vestibular function tests may support diagnostic refinement and treatment planning in patients who do not (yet) completely fulfill the criteria for VM nor for MD. If either the vHIT or caloric test is abnormal, a diagnosis of MD is more likely. The optimal sensitivity and specificity to differentiate between MD and VM was found with a UW threshold of 34%. However, in case of an abnormal vHIT result, the caloric test can be considered redundant.

## Data Availability

The raw data supporting the conclusions of this article will be made available by the authors, without undue reservation.
