# Supplementary material for: Vestibular function tests are helpful in differentiating between Menière’s disease and vestibular migraine
Source: Front Neurol. 2025 Aug 7;16:1569247. doi: 10.3389/fneur.2025.1569247 (PMC12367752; doi:10.3389/fneur.2025.1569247)
Supplement: Supplementary file 1 [file Table_1.pdf]

frontiers

Supplementary Material

Supplementary table 1. Demographics and symptoms of patients with a double diagnosis and patients with bilateral MD.

	MDVM<br>n = 5	pMDpVM<br>n = 67	Bilateral MD<br>n = 22
Gender, n female (%)	3 (60.0%)	48 (71.6%)	9 (40.9%)
Age at presentation, years (mean±SD)	57.0±8.2	50.6±14.4	67.3±12.1
Duration of symptoms, years (median (IQR))	20.0 (29.5)<br>n = 5	3.0 (6.0)<br>n = 62	19.0 (19.0)<br>n = 21
History of migraine, n (%)	5 (100.0%)<br>n = 5	34 (51.5%)<br>n = 66	2 (9.1%)<br>n = 22
Ictal headache n, %	4 (80.0%)<br>n = 5	26 (40.0%)<br>n = 65	7 (35.0%)<br>n = 20
Aural symptoms (any) n, %	5 (100.0%)	67 (100.0%)	22 (100.0%)
• Subjective hearing loss n, %	4 (80.0%)<br>n = 5	44 (68.8%)<br>n = 64	21 (95.5%)<br>n = 22
• Tinnitus n, %	5 (100.0%)<br>n = 5	49 (77.8%)<br>n = 63	18 (85.7%)<br>n = 21
• Aural fullness n, %	4 (80.0%)<br>n = 5	52 (78.8%)<br>n = 66	15 (75.0%)<br>n = 20

MDVM = Menière's disease and vestibular migraine; pMDpVM = probable Menière's disease and probable vestibular migraine; MD = Menière's disease; n = number of patients; SD = standard deviation; IQR: interquartile range.
